# Cervid herpesvirus 2 and not *Moraxella bovoculi* caused keratoconjunctivitis in experimentally inoculated semi-domesticated Eurasian tundra reindeer

**DOI:** 10.1186/s13028-017-0291-2

**Published:** 2017-04-24

**Authors:** Morten Tryland, Javier Sánchez Romano, Nina Marcin, Ingebjørg Helena Nymo, Terje Domaas Josefsen, Karen Kristine Sørensen, Torill Mørk

**Affiliations:** 1 Arctic Infection Biology, Department of Arctic and Marine Biology, UiT-The Arctic University of Norway, POBox 6050, Langnes, 9037 Tromsø, Norway; 2 Norwegian Veterinary Institute, POBox 6050, Langnes, 9037 Tromsø, Norway; 3 Vascular Biology Research Group, Department of Medical Biology, Faculty of Health Sciences, UiT-The Arctic University of Norway, Tromsø, Norway; 4 Clinique vétérinaire de l’abbatiale, 14 bis Rue Thibaut, 52220 Montier En Der, France; 5 Faculty of Bioscience and Aquaculture, Nord University, Bodø, Norway

**Keywords:** Alphaherpesvirus, Eye disease, IKC, Moraxella, Ophthalmology, Reindeer, Wildlife

## Abstract

**Background:**

Infectious keratoconjunctivitis (IKC) is a transmissible disease in semi-domesticated Eurasian reindeer (*Rangifer tarandus tarandus*). It is regarded as multifactorial and a single causative pathogen has not yet been identified. From clinical outbreaks we have previously identified Cervid herpesvirus 2 (CvHV2) and *Moraxella bovoculi* as candidates for experimental investigations. Eighteen reindeer were inoculated in the right eye with CvHV2 (n = 5), *M. bovoculi* (n = 5), CvHV2 and *M. bovoculi* (n = 5) or sterile saline water (n = 3; controls).

**Results:**

All animals inoculated with CvHv2, alone or in combination with *M. bovoculi*, showed raised body temperature, increased lacrimation, conjunctivitis, excretion of pus and periorbital oedema; clinical signs that increased in severity from day 2 post inoculation (p.i.) and throughout the experiment, until euthanasia 5–7 days p.i. Examination after euthanasia revealed corneal oedema, and three animals displayed a corneal ulcer. CvHV2 could be identified in swab samples from both the inoculated eye and the control eye from most animals and time points, indicating a viral spread from the inoculation site.

**Conclusions:**

This study showed that CvHV2 alone and in combination with *M. bovoculi* was able to cause the characteristic clinical signs of IKC in reindeer, whereas inoculation of *M. bovoculi* alone, originally isolated from a reindeer with IKC, did not produce clinical signs. Previous studies have suggested that herding procedures, animal stress and subsequent reactivation of latent CvHV2 infection in older animals is a plausible mechanism for IKC outbreaks among reindeer calves and young animals in reindeer herds. However, further studies are needed to fully understand the infection biology and epidemiology associated with IKC in reindeer.

## Background

**Fig. 1:**
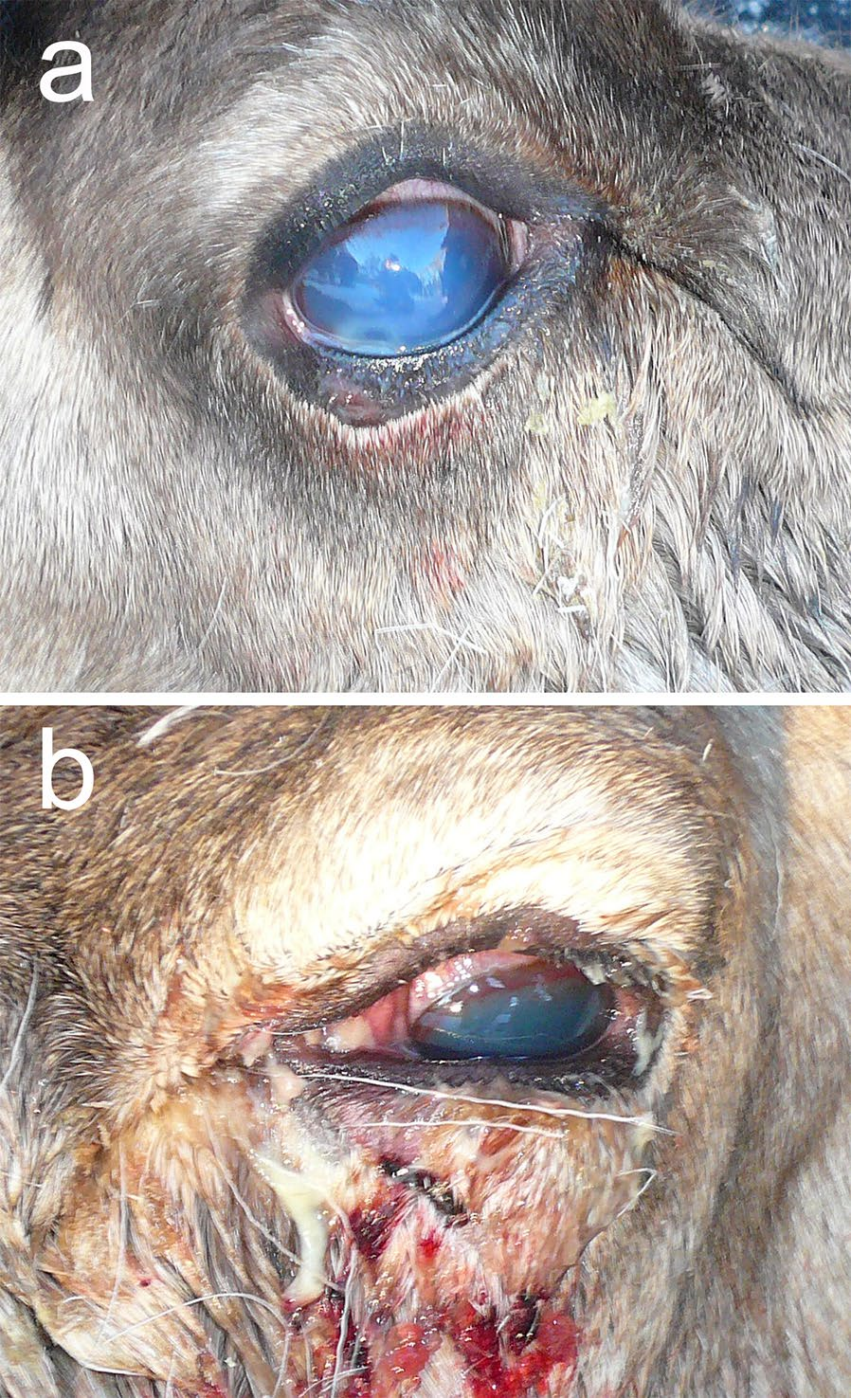
Infectious keratoconjunctivitis (IKC) during an outbreak among free-ranging Eurasian semi-domesticated tundra reindeer (*Rangifer tarandus tarandus*): **a** corneal oedema indicated by an opaque and discolored cornea. **b** Severe grade of IKC with panophthalimitis, oedema and haemorrhage. This condition often involves corneal ulcer and may lead to permanent blindness

Infectious keratoconjunctivitis (IKC) has been reported in semi-domesticated Eurasian tundra reindeer (*Rangifer tarandus tarandus*) in Fennoscandia for more than a century [1], causing outbreaks among calves of the year (<1 year) and young animals particularly [2–4]. The first sign of the disease is increased lacrimation observed as an untidy and miscoloured periocular hair coat followed by conjunctivitis and increasing periorbital and corneal oedema. The oedema is giving the eye an opaque, and whitish to bluish appearance (Fig. 1a), which is how IKC is usually recognized in the field by reindeer herders. In the absence of spontaneous healing, the infection progresses with an increasing severity of conjunctivitis and oedema, corneal ulceration, and panophthalmitis leading to permanent blindness (Fig. 1b). For reindeer, that spend most of the year free-ranging and unattended, this disease represents an important animal welfare issue as well as a potential source of economic loss for the herders [2, 4].

In spite of long-term awareness of IKC in reindeer, a causative agent has not yet been identified and IKC in reindeer is regarded as a multi-factorial disease, similar to infectious bovine keratoconjunctivitis (IBK), commonly referred to as “pinkeye” in cattle. The Gram-negative bacterium *Moraxella bovis* is suggested as the causative agent of IBK [5], although recent studies have shown that *Moraxella bovoculi*, originally identified in 2002 and shown to be distinct from *M. bovis*, may also be associated with IBK [6, 7]. A disease similar to IKC has also been documented in sheep and goats [8, 9], as well as in a wide range of wild animal species, such as moose (*Alces alces*), mule deer (*Odocoileus hemionus*), ibex (*Capra ibex*), chamois (*Rupicapra rupicapra*), roe deer (*Capreolus capreolus*) and red deer (*Cervus elaphus*). Many different infectious agents have been associated with the disease in the different species [10–14], of which the majority have been bacteria, including known potential pathogens such as *Mycoplasma* spp.

Similarly, many different bacteria have been isolated from reindeer with IKC, such as *M. bovoculi*, *M. ovis*, *Pasteurella multocida*, and *Trueperella pyogenes* [2–4, 15, 16]. In a recent study of reindeer in Norway, Sweden and Finland, analyses of swab samples from the conjunctiva of clinically healthy reindeer and of reindeer with clinical IKC, showed that *M. bovoculi* was the bacterial agent that was most prevalent [17].

However, during an outbreak of IKC in reindeer in Troms County, Norway, the reindeer alphaherpesvirus, cervid herpesvirus 2 (CvHV2), was identified as the primary infectious agent based on the finding of this virus during the early clinical stages of the disease. Bacteria such as *M. bovoculi* seemed to dominate during later stages of IKC along with declining virus titers from the swab samples of the conjunctiva of affected animals [4].

In addition, environmental factors such as stress associated with gathering, corralling, handling and transport, reduced body condition or emaciation, exposure to UV light, and a dry and dusty environment may impact the pathogenesis of IKC in reindeer [2, 18, 19]. During natural outbreaks of IKC in reindeer, it is difficult to gain an overview of which potential pathogens are involved. It is also challenging to conduct a controlled sampling regime and to register the onset and development of disease. In addition, it is difficult to control environmental factors. In this study, reindeer calves <1 year old were inoculated with CvHV2 and *M. bovoculi,* which are considered the two most relevant candidates for IKC [4, 17]. The aim of this study was to investigate if experimental inoculation with CvHV2, *M. bovoculi*, or a combination of the two agents, was able to cause clinical IKC in reindeer.

## Methods

### Animals

Semi-domesticated Eurasian tundra reindeer (n = 27) calves, approximately 11 months old, were gathered from their mountain pastures. They were corralled and physically restrained for ID-tagging (plastic collar) and sampling to check for previous exposure to CvHV2 and *M. bovoculi*. Blood was obtained with a vacutainer system from the jugular vein and serum prepared by centrifugation and stored at −20 °C. For virological investigation, sterile cotton swabs were used to sample the conjunctiva bilaterally, the nostrils and the vagina (females). Swabs were subsequently placed in 1.8 ml cryotubes with 800 µl of Eagle’s minimum essential medium (EMEM) containing antibiotics (10,000 U/ml penicillin and 10 mg/ml streptomycin; 1 ml/l of gentamicin, 50 mg/ml and 10 ml/l of amphotericin B 250 μg/ml; EMEMab 10 ml/l) and stored at −80 °C. For bacteriological investigation, a swab (Transwab^R^, Medical Wire & Equipment, Wiltshire, UK) was introduced into both conjunctivae, rubbed gently against the mucosal lining and placed in its transport medium container. Blood samples were tested for antibodies against CvHV2. Swab samples from the conjunctiva and nostrils were tested for the presence of CvHV2 DNA (nested polymerase chain reaction [PCR]), whereas swab samples from conjunctiva were tested for *M. bovoculi* (cultivation), as described below. Based on the test results, 21 animals (5 females and 16 males) with no indication of previous exposure to CvHV2 or *M. bovoculi* were recruited to the study. The animals were corralled together in a fence (see below) for a habituation period of four weeks before inoculation. The inoculation experiment lasted for a total of 15 days.

All animals were chemically immobilized and weighed twice during the study, i.e. on day 0 (inoculation) and on the day of euthanasia. Animals were immobilized by darting (Dan-Inject JM Special; Dan-Inject ApS, Børkop, Denmark) using a combination of medetomidine (Zalopine^®^ 10 mg/ml, Orion Corporation Animal Health, Espoo, Finland) and ketamine (Ketalar^®^ 50 mg/ml, Pfizer AS, Oslo, Norway) at a fixed ratio of 1:5 (mg:mg) medetomidine:ketamine. When the inoculation process was completed, sedation was reversed by intramuscular injection of atipamezole (Antisedan™; Atipamezole 5 mg/ml, Zoetis, UK) at a fixed ratio of 5:1 atipamezole:medetomidine and animals were monitored until they were standing. Despite using recommended drugs and doses being reduced to one-third of those recommended for semi-domesticated reindeer [20], three reindeer (one female, two males) died from acute shock-like complications. Similar complications did not occur during the second round of chemical immobilization using the same drugs and doses. When chemically immobilized, body temperature was measured with a digital clinical thermometer (Fluke Thermometer 51/52 II; Fluke Norge, Oslo, Norway), with a sensor positioned 30 cm inside the rectum. Pulse rate and arterial oxyhemoglobin (SpO_2_) was measured with a handheld pulse oximeter (Masimo RAD 57; Masimo International Sàrl, Neuchatel, Switzerland) with the clip-sensor applied to the tongue. Respiratory rate was recorded by counting breathing movements of the chest using a stopwatch.

Animals were euthanized at different time points during the experiment using a captive bolt stunning gun followed by bleeding of the jugular vein. Animals R19 (CvHV2-group) and R16 (*M. bovoculi* group) were euthanized on day 2 and 3 respectively, due to fracture of the femur. Animals in the two groups inoculated with CvHV2 (alone or in combination with *M. bovoculi*) were euthanized according to defined animal welfare end-points on days 3–7 post inoculation (p.i.) (Table 1). Remaining animals of the *M. bovoculi* group were euthanized on day 6 (n = 1) and day 13 (n = 3) p.i., whereas the control animals (n = 3) were euthanized on day 15 p.i. Animals with no other clinical symptoms besides those observed in the eyes were sampled in the animal experimental facility, whereas animals with trauma and animals that experienced respiratory distress upon chemical immobilization were subjected to a full necropsy in an attempt to determine the cause of these unexpected complications.

**Table 1:** Clinical signs from the right eye of 10 semi-domesticated Eurasian tundra reindeer (*Rangifer tarandus tarandus*) after inoculation with cervid herpesvirus 2 (CvHV2; n = 5) or a combination of CvHV2 and *Moraxella bovoculi* (n = 5) (data from animals inoculated with *M. bovoculi* alone or from control animals are not shown)

Inoculum	ID	Day 0	Day 1	Day 2	Day 3	Day 4	Day 5	Day 6	Day 7
CvHV2	R4	None		Pus (2)^a^ P-oedema (1)^b^ Conjunctivitis	Pus (2) P-oedema (3)	Lacrimation P-oedema (3)	Pus (3) P-oedema (3) Corneal oedema T: 40.3	Pus (3) P-oedema (3)	*Pus (3)* *P-oedema (3)* *Conjunctivitis* *Corneal oedema* *T: 39.8*
	R5	None		Pus (1) P-oedema (1)	Pus (3) P-oedema (2)	Pus (3) P-oedema (3)	*Pus (3)* *P-oedema (3)* *Corneal oedema* *Corneal ulcer: 12* *mm* *T: 40.5*		
	R10	None		Pus (3) P-oedema (1) Conjunctivitis	Pus (3) P-oedema (2)	Pus (3) P-oedema (3)	*Pus (3)* *P-oedema (3)* *Conjunctivitis* *Corneal oedema* *T: 40.0*		
	R19	None		*Lacrimation* *Pus (1)* *P-oedema (1)* ^c^					
	R21	None		P-oedema (1)	Lacrimation Pus (2) P-oedema (2)	Lacrimation Pus (2) P-oedema (2)	Pus (2) P-oedema (2) Corneal oedema Corneal ulcer: 4 mm	*Pus (3)* *P-oedema (3)* *Corneal oedema* *Corneal ulcer: 10* *mm* *T: 40.1*	
CvHV2 and *M. bovoculi*	R1	None	Pus (1)	Lacrimation Pus (1) P-oedema (1) Red sclera	Lacrimation Pus (2) P-oedema (2)	Pus (2) Lacrimation P-oedema (2)	Pus (3) P-oedema (3) Corneal oedema T: 40.4	Pus (3) P-oedema (3)	*P-oedema (3)* *Conjunctivitis* *Corneal oedema* *Corneal ulcer: 3* *mm* *T: 39.1*
	R3	None		Lacrimation P-oedema (1)	Pus (3) P-oedema (3)	Pus (3) P-oedema (3)	P-oedema (3) Adherent pus Blue cornea T: 40.0	*Pus (3)* *Conjunctivitis* *Corneal oedema* *T: 39.8*	
	R7	None		Pus (1)	Pus (2) Lacrimation P-oedema (2)	Pus (2) Lacrimation P-oedema (2)	*Pus (3)* *P-oedema (3)* *Conjunctivitis* *Corneal oedema* *T: 40.3*		
	R13	None		Pus (1) Lacrimation Red sclera P-oedema (2)	Pus (3) Lacrimation P-oedema (2)	*Pus (3)* *P-oedema (3)* *Conjunctivitis* *Corneal oedema* *T: 40.4*			
	R17	None	Dry cornea	Pus (1) Lacrimation P-oedema (2)	*Pus (3)* *P-oedema (2)* *T: 40.9*				

Clinical signs are indicated, relative to the day of inoculation (day 0). Observations during day 1, day 3, day 4 and day 6 were conducted without handling the animals, whereas all animals were captured (physical restraint) and sampled at day 2 and day 5

The time of euthanasia for each animal is indicated with italic text

^a^Pus: (1) small amounts (clots), (2) large amounts, and (3) huge amounts of pus, adherent to eyelids/skin and covering the eye

^b^P-oedema: periorbital oedema: (1) slight swelling of conjunctiva, (2) severe oedema, and (3) extensive oedema, periorbital tissues covering the eyeball

^c^Euthanized due to trauma

### Animal facility, feeding and handling

Animals were introduced to an outdoor field research facility on May 6th, 2014. The corral consists of one large pen (approx. 100 × 80 m) and four smaller pens (approx. 50 × 50 m), all separated from each other and from the surrounding areas by 2.5 m high steel wire fences. The ground inside the pens was covered with a deep layer of snow in the beginning, but due to snow melting and greening in the spring, fresh grass pasture became available. Animals were ID-tagged with an ear tag and by spraying a number on the fur on both sides of the body to ensure easy identification. They were corralled together for the first four weeks (habituation period) to get used to the facility, the feed and the presence of people.

The animals were fed lichen (*Cladina* spp.), which was gradually replaced by commercial pelleted feed for reindeer (Reinsdyrfôr, Felleskjøpet, Trondheim, Norway). 1 week before inoculation and throughout the remaining experiment, the animals were fed pellets ad libitum, supplemented with lichen. Fresh water was provided and changed on a daily basis. Body condition, evaluated by visual inspection and palpation, varied from medium to lean, but increased during the habituation period. On the day of inoculation, the animals weighed 37–59 kg (mean 49.1 kg) and were separated into four groups without further contact (Control, CvHV2, *M. bovoculi* and CvHV2/*M. bovoculi*).

During the entire study, the animals were fed and inspected three times a day by veterinarians licenced for conducting animal experiments. A record was kept describing the behaviour of each animal, condition, food intake and interactions between animals as well as development of clinical signs.

Except for day 0 (inoculation) and on the day of euthanasia at varying time points p.i. when animals were chemically immobilized, all other close inspections and sampling were conducted during physical restraint. However, on days when animals were not handled, the evaluation and development of clinical signs was observed at a distance of a few metres with binoculars.

### Preparation of inocula

CvHV2 was originally isolated from the conjunctiva of a reindeer with clinical IKC [4]. The virus was propagated in Madin-Darby bovine kidney (MDBK) cells (ATCC CCL22) in EMEM supplemented with 10% horse serum (LGC Standards, Middlesex, UK). Virus cultures were harvested when more than 90% of the cells showed cytopathic effect (CPE). Cellular debris was removed by centrifugation (7800*g*, 30 min) and viral particles in the supernatant were pelleted through a 30% sucrose cushion (20,000 g, 1 h), re-suspended in 5 ml PBS, filtered through a 0.45 μm cellulose filter (Merck Millipore, Darmstadt, Germany) and frozen at −80 °C. The virus titre of the inoculum was 5.8 × 10^6^ TCID_50_/ml.

The *M. bovoculi* strain used for inoculation was originally cultivated from a swab sample from the conjunctiva of a Norwegian reindeer with clinical IKC in 2014. The colonies were 2–3 mm in diameter, yellow/grey, round, with a shiny “fat” appearance, and revealed beta-haemolysis after 24 h growth on blood agar. The colonies grew at 37 °C, but only in aerobic conditions (without CO_2_). The bacteria were Gram negative with a coccoid form, and the catalase and oxidase tests were positive. Potential virulence factors were not investigated. The isolate was verified as *M. bovoculi* by 16s rRNA sequencing (data not shown). After storage (−80 °C), the isolate was cultivated on 5% sheep blood agar at 37 °C under aerobic conditions for 24 h prior to inoculation. The bacterium underwent a total of four in vitro passages (from isolation to inoculation).

### Inoculation

While the animals were sedated, both eyes were photographed and checked for general clinical signs and for corneal ulcers using the fluorescein test (Fluoresceinnatrium Minims 20 mg/ml; Chauvin, Surrey, UK). Disposable coats and gloves were used by all persons involved in the inoculation and sampling of the animals. For any intervention with the animals (feeding, inoculation, sampling), the control group was visited before the inoculated animals.

Prior to inoculation and at each of the following inspections, eyes were photographed, sampled (swab) and checked for corneal ulcers using fluorescein, followed by thorough flushing of the eyes with sterile water. Local anaesthesia (Oxibuprokain Minims 4 mg/ml; Chauvin) was applied in the right eye 4 min prior to a mild rubbing of the conjunctival mucosa with sterile sandpaper (grade 60).

The virus was inoculated by introducing a small sterile cotton cushion, soaked in PBS/CvHV2, to the conjunctiva under the lower eyelid and keeping it there for 4 min. *M. bovoculi* was inoculated by picking a colony of *M. bovoculi* from the agar plate with a sterile cotton stick and spreading it onto the rubbed area of the conjunctiva. Bacterial colonies from the same plate were subsequently reseeded onto new agar plates to check for viability. These procedures for viral and bacterial inoculations were conducted to allow access of virus and bacteria in excess to slightly scarified mucosal cells of the conjunctiva. When inoculating both CvHV2 and *M. bovoculi* in the same eye, the virus was inoculated first, followed by the bacterium after 10 min. For the animals in the control group, a sterile cotton stick was soaked in sterile water and applied onto the rubbed conjunctiva. The left eye was inspected as described above, but not further manipulated, and served as a control for each animal (all groups).

### Bacterial cultivation

Swab samples from the conjunctiva of both eyes, obtained on the day of inoculation (Day 0) and every second day after inoculation, were subjected to cultivation on a 5% sheep blood agar plate. The plates were incubated aerobically at 37 °C and inspected after 24 and 48 h. Bacterial colonies that were yellow to grey of colour, 2–3 mm in diameter, with a shiny “fat” appearance and were beta-haemolytic, were suspected to be *Moraxella* spp. Such colonies were sub-cultured for purity and further characterized (morphology, Gram staining, catalase, oxidase). Selected isolates were subsequently identified to species level (score > 2) by MALDI-TOF mass spectrometry (Microflex; Bruker UK Limited, Coventry, UK).

### DNA extraction, PCR and sequencing

To verify that the animals did not harbour CvHV2 viral particles in the conjunctival mucosa prior to the experiment, DNA was extracted from swabs obtained from the conjunctival mucosa (QIAamp^®^ DNA Mini Kit, Hilden, Germany) with a mean output of 8.1 ng/µl (SD = 8.0) and a nested PCR was conducted as described previously [21]. The inner primer set amplified a 294 bp region of the *UL27* gene encoding glycoprotein B (gB), which is a highly conserved gene region among ruminant alphaherpesviruses [22]. CvHV2 (strain Salla 82) [23] and BoHV1 [24] were used as positive controls. PCR amplicons were separated by agarose gel electrophoresis and visualized with ethidium bromide (Sigma-Aldrich Norway AS, Oslo, Norway).

A quantitative real-time Taqman Probe based PCR (qPCR), amplifying a 95 bp fragment in a different region of the *UL27* gene as compared to the nested PCR and shown previously to detect Rangiferine herpesvirus (now designated CvHV2), was performed as described previously [25]. The PCR was conducted with two slight modifications; the annealing temperature was reduced to 58.9 °C (following thermal gradient analysis) and the volume of nuclease-free water was increased from 4.0 to 4.5 µl for a total reaction volume of 25 µl. Samples were run in duplicates. A positive control (CvHV2, used as inoculum) [4], a negative control (DNA extracted from muscular tissue of a CvHV2 seronegative control animal), and a non-template control containing all PCR-components except DNA were included on each plate. This technique was used to analyse all eye swab and plasma samples collected from day 0 and throughout the experiment.

PCR amplicons were prepared for nucleotide sequencing by enzymatic removal of unused dNTP and primers (ExoSAP-IT™; Amersham Pharmacia Biotech, Sweden), after which sequencing was conducted (BigDye^®^ Terminator v3.1 cycle sequencing kit; Applied Biosystems, Norway) in an Applied Biosystems 3130 XL Genetic Analyzer (Applied Biosystems).

### Serology

Serum samples were investigated for anti-alphaherpesvirus antibodies using a commercial bovine enzyme-linked immunosorbent assay (ELISA; gB Blocking, LSI Laboratoire Service International, Lissieu, France) based on BoHV1 glycoprotein B (gB) as an antigen. The kit was previously validated against a virus neutralization test (VNT) for analyzing reindeer serum samples for anti-CvHV2 antibodies [26]. All serum samples were tested in duplicate and evaluated against bovine (provided with the kit) and reindeer [27] positive-control sera.

## Results

### Clinical signs

A thorough clinical examination, including body temperature measurement, was conducted on each animal when they were physically or chemically immobilized, i.e. upon arrival to the fence, on the day of inoculation one month later (day 0), and on days 2, 5, 7, 13 and 15 p.i., as well as upon euthanasia, if euthanized outside this schedule.

Body temperature (n = 18) at the time of inoculation (day 0) varied from 37.8 to 39.5 **°**C (mean 39.0 **°**C, SD 0.05). On day 5 p.i., the mean body temperature for all inoculated animals (except the controls) had increased to 39.8–40.6 **°**C (mean 40.2 **°**C), and decreased again over the following days. Mean temperature also increased for the control animals (n = 3), from 39.0 **°**C (day 0) to 39.9 **°**C (day 7), after which it decreased to 39.6 **°**C (day 10) and 39.3 **°**C (day 15).

**Fig. 2:**
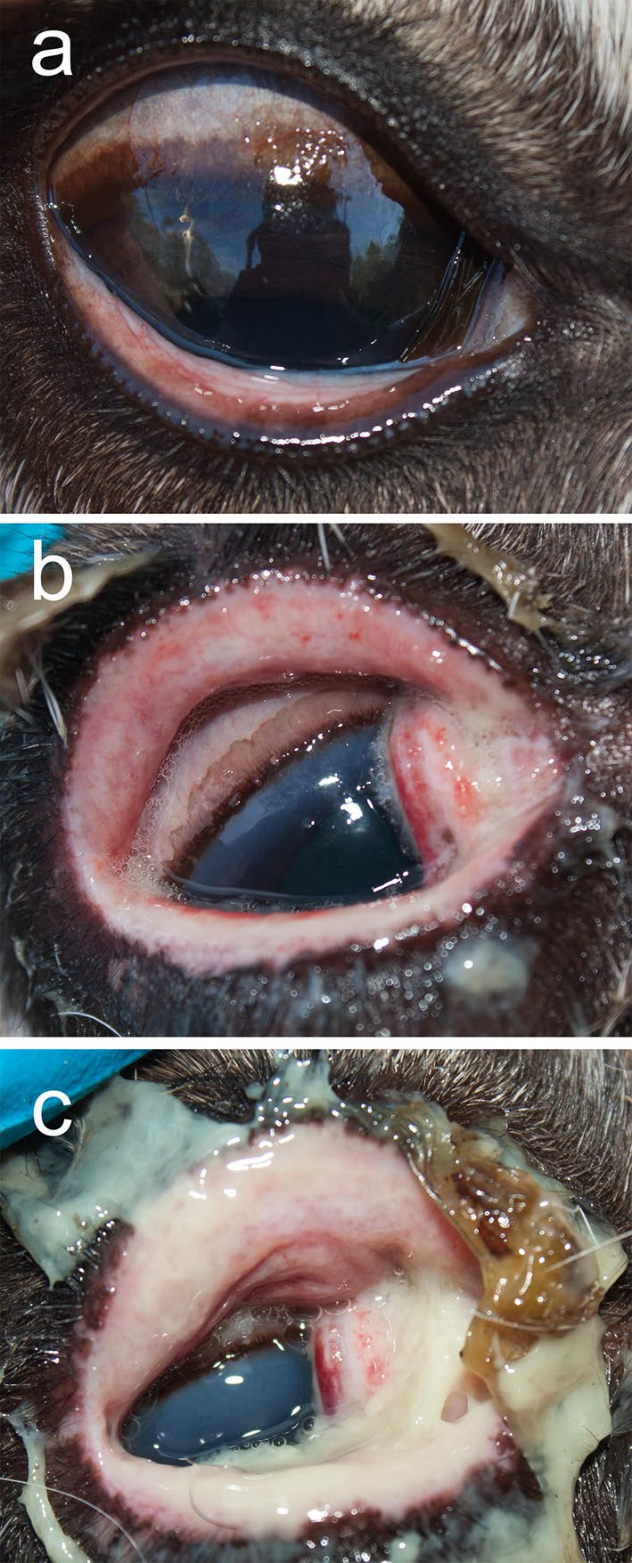
Semi-domesticated Eurasian tundra reindeer (R21) experimentally inoculated with reindeer alphaherpesvirus (cervid herpesvirus 2; CvHV2). **a** Day 0, i.e. the day of inoculation, **b** day 5 post inoculation (p.i.), and **c** day 6 p.i.

In the CvHV2 group, no clinical signs were observed on day 1 (Table 1). On day 2, the inoculated eyes of all animals in this group displayed conjunctival oedema, four of the five animals having purulent exudate and two having hyperaemia of the conjunctiva (3). From day 3 to day 5, the severity of the oedema typically increased from mild (grade 1) to severe (grade 3), involving the whole periorbital region by day 5. Similarly, the suppuration increased from mild (grade 1) to severe (grade 3) (Fig. 2). Upon inspection on day 5, two animals (R5 and R10) were euthanized, showing corneal oedema, high grades of periorbital oedema and suppuration, as well as a slightly raised body temperature, 40.5 and 40.0 °C, respectively. Similar clinical signs were observed for R21 and R4, which were euthanized on day 6 and 7, respectively (Fig. 2). Animals R5 and R21 had at the time of euthanasia developed a corneal ulcer, by day 5 and day 6, respectively.

**Fig. 3:**
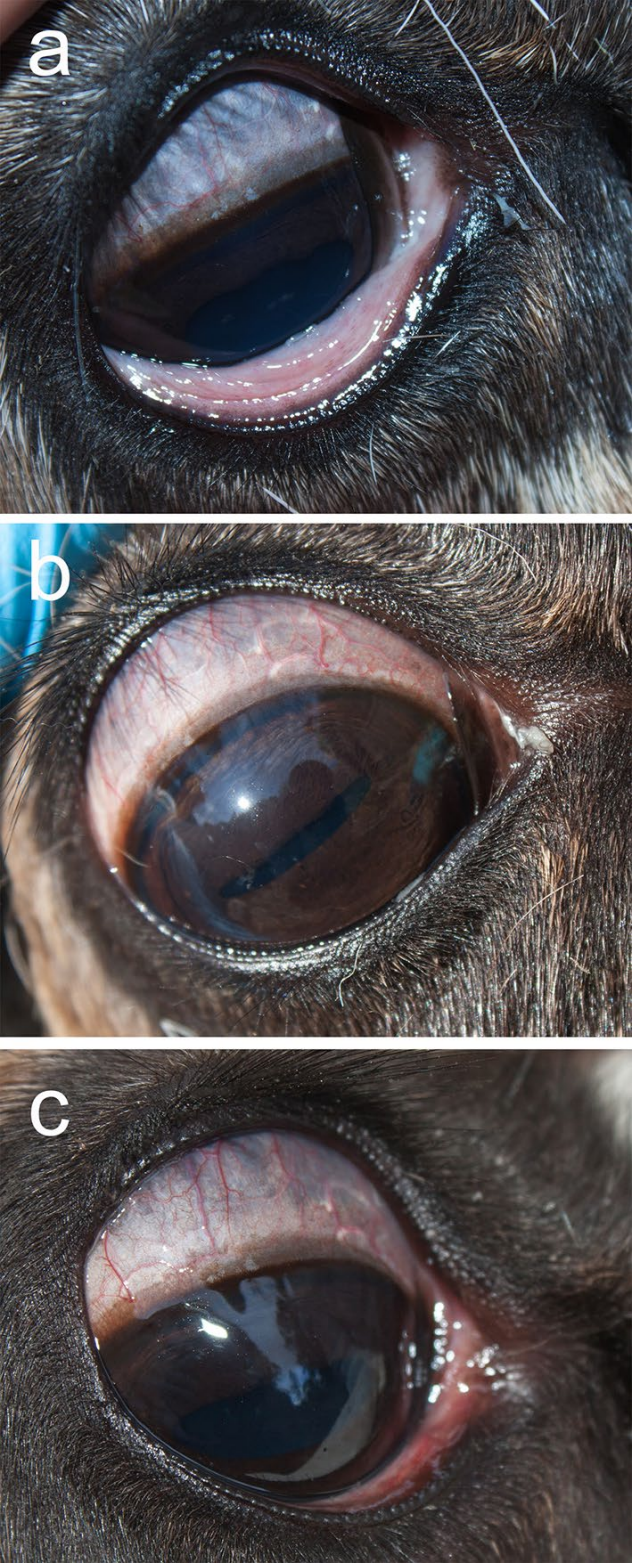
Semi-domesticated Eurasian tundra reindeer (R15) inoculated with *Moraxella bovculi.* **a** Day 0, i.e. the day of inoculation, **b** day 5 post inoculation (p.i.), and **c** day 10 p.i.

In the *M. bovoculi* group, a grey or whitish exudate was observed associated with the lower eyelid of the inoculated right eye in two animals (R6 and R8) some hours after inoculation (day 0) and early the following day (day 1), which thereafter disappeared. No clinical signs were observed in this group throughout the remaining period of the experiment (Fig. 3).

**Fig. 4:**
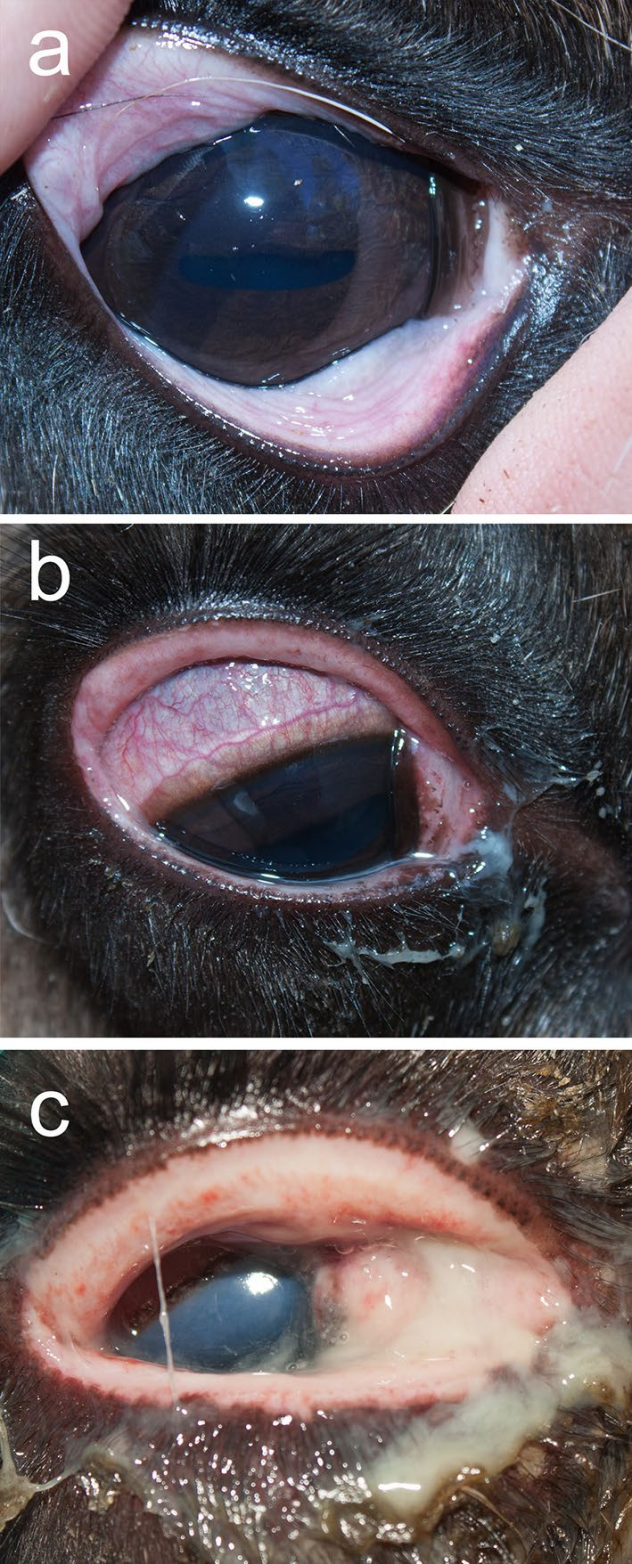
Semi-domesticated Eurasian tundra reindeer (R1) inoculated with reindeer alphaherpesvirus (cervid herpesvirus 2; CvHV2) and *Moraxella bovculi*. **a** Day 0, i.e. the day of inoculation, **b** day 2 post inoculation (p.i.), and **c** day 5 p.i.

The clinical signs observed in the group receiving both CvHV2 and *M. bovoculi* were indistinguishable from those described for the CvHV2 group (Table 1). All animals in this group, except the one euthanized on day 3 (R17), showed suppuration and developed conjunctivitis, and corneal and periorbital oedema (Fig. 4). One animal (R1) developed a corneal ulcer.

No clinical signs of disease were recorded in the control animals (R2, R18, R20) throughout the experiment (day 0–15).

### Bacteriology

On day 0, prior to inoculation, a few colonies of *M. bovoculi* were cultured from the conjunctiva of two animals (R6 and R8) from the group to be inoculated with *M. bovoculi*, and from another two animals (R1 and R17) from the group to be inoculated with CvHV2 and *M. bovoculi*. These isolates were all identified using the MALDI-TOF mass spectrometry. During the experiment, *M. bovoculi* was, at different time points, cultivated from all five animals inoculated with the bacterium. In the group inoculated with CvHV2 and *M. bovoculi*, the bacterium was cultivated from three of five animals. In the group inoculated with CvHV2 alone, *M. bovoculi* was cultivated from four of five animals. No *M. bovoculi* was isolated from the control animals. In most cases, *M. bovoculi* was found with few colonies in a mixed culture.

### PCR

In spite of being sero-negative, CvHV2-specific DNA was detected in one eye swab (nested PCR; R21) before the experiment, and this animal was allocated to the CvHV2 group. No CvHV2-specific DNA was detected from conjunctival swab samples from this animal or any other swab samples (both eyes, all animals) taken on day 0 of the experiment, i.e. prior to inoculation. Of the animals inoculated with CvHV2, alone or in combination with *M. bovoculi*, CvHV2 specific DNA was detected (qPCR) in all samples from both inoculated and control eyes, from day 2 until the last sampling of each animal (euthanasia). There were two exceptions: CvHV2 could not be detected in the swab sample from the right (inoculated) eye of animal R3 on day 5 (the day before euthanasia, though detected again upon euthanasia, day 6), nor from the left eye (not inoculated) of animal R21 on day 6 (the day of euthanasia).

From the animals inoculated with only *M. bovoculi*, CvHV2-specific DNA was detected in one sample from the left eye of animal R6 on day 6, but besides this, all samples from animals in this group and the control group were negative for CvHV2. Viral DNA was detected in the plasma of one animal (R7) from the CvHV2-inoculated group on day 2 but not in the plasma of this animal at later time points, and not from plasma samples from any other animal during the experiment.

### Serology (CvHV2)

All animals selected for the study were sero-negative for CvHV2 upon arrival and on the day of inoculation (day 0). Animals R21 and R4 from the group inoculated with CvHV2 seroconverted on day 6 and 7, respectively, and animal R1 from the group inoculated with CvHV2 and *M. bovoculi* seroconverted on day 7.

## Discussion

All animals inoculated with CvHV2, alone or in combination with *M. bovoculi* (Figs. 2 and 4) developed clinical signs characteristic of IKC [2, 4] with a quick onset (day 2) and rapid progression from day 2 to days 5/6/7 (euthanasia). The animals inoculated with *M*. *bovoculi* alone, and the control animals, remained healthy throughout the experiment. These unambiguous clinical results, generated from an experimental inoculation of one-year-old reindeer with all members of each animal group displaying similar responses, provide powerful support of previous findings during IKC outbreaks [4], that CvHV2 is capable of causing the clinical picture that is characteristic for IKC in reindeer.

The titres of the inoculum of virus and bacteria in this study were probably high as compared to viral and bacterial transmissions under natural conditions, but nevertheless, for the virus, in line with previous reported inoculations of BoHV1 in cattle [28]. It is, however, important to keep in mind that both the virus and the bacterium are replicative agents that, once the infection is established, will multiply in permissive cells, whereas virus and bacteria in excess will be washed away from the surface linings of the eye, rendering the inoculum titres less important.

We gently rubbed a part of the conjunctival mucosa of the lower eye lid to make sure that the infectious agents had access to permissive cells upon inoculation, but we chose not to conduct scarified corneal inoculations as has been done in similar experiments using cattle and *Moraxella* spp. [29]. The rubbing may be somewhat similar to what reindeer may experience when corralled and exposed to sand and dust in the pen. Thus, we think the conditions under which this experiment was carried out were in many ways close to a natural setting, including the fact that animals were young (<1 year) at the time of inoculation, and that they had been exposed to stress by being gathered from their mountain pastures, transported and corralled, and by being exposed to people and handling, all resembling natural herding conditions.

In IBK in cattle, the pathogenicity of *M. bovis* is based on the expression of a pilin protein for attachment [30] and a cytotoxin that damages the corneal epithelial cells [31]. However, *M. bovoculi* has also been associated with IBK. In a retrospective study of *Moraxella* spp. isolated from IBK outbreaks in 282 herds in 30 states of the USA, 701 were identified as *M. bovoculi* and 295 isolates as *M. bovis* [32]. It has also been shown that *M. bovoculi* isolates from cattle may have both putative cytotoxin [33] and pilin [34] genes, similar to those that have been identified in *M. bovis*. However, a randomized blind challenge study to assess the association between *M. bovis* and *M. bovoculi,* and IBK in 31 dairy calves, revealed that nine out of 10 calves inoculated with *M. bovis* developed corneal ulcers consistent with IBK, whereas none of the 10 calves inoculated with *M. bovoculi* did, although the authors stated that the pathogenicity of this particular *M. bovoculi* strain was not yet characterized [29]. This was also the situation for the *M. bovoculi* isolate used in our study, although it was originally isolated and identified as the bacterium that was dominating the bacterial growth from a swab sample obtained from the conjunctiva of a reindeer with clinical signs of IKC. Large genetic differences have recently been reported between isolates of *M. bovoculi* from eyes of cattle with IBK as compared to isolates from the nasopharynx of asymptomatic cattle, suggesting that certain genetically distinct strains of *M. bovoculi* are associated with IBK in cattle [35], which may also be the case for IKC in reindeer.

Throughout our study period, *M. bovoculi* was cultivated from eight of 10 animals that had been inoculated with the bacterium alone or in combination with CvHV2, but no clinical signs were associated with these findings in those inoculated with *M. bovoculi* alone. This might indicate that the isolate of *M. bovoculi* used for inoculation was not pathogenic, or that the duration of the bacterial infection was too short to produce clinical signs. *M. bovoculi* was also cultivated from four animals prior to inoculation (day 0), and from four animals inoculated with CvHV2 alone, from the right eye of all four animals and from both eyes of one individual. This may indicate the transmission of bacteria from other individuals, which means that *M. bovoculi* was transferred from inoculated animals into the other corrals. However, the fact that *M. bovoculi* was not cultivated from any of the control animals at any time (day 0–day 13 p.i.) indicated that *M. bovoculi* was not introduced but rather sporadically present in apparently healthy reindeer without ocular disease. The bacterium may therefore be a commensal in the conjunctiva of reindeer as also indicated from previous bacteriological screenings [17]. Since serology for antibodies against *M. bovoculi* prior to the inoculation was not conducted, it can be argued that a lack of clinical signs in the animals inoculated with *M. bovoculi* alone may be due to a pre-existing immunity against the bacterium, but it remains uncertain if presence of the bacterium on mucosal membranes such as conjunctiva and nasopharynx would elicit a detectable humoral immune response.

Animals R1 (CvHV2 and *M. bovoculi*), and R4 and R21 (CvHV2) had antibodies against CvHV2 on the day of euthanasia (day 6 and 7, respectively), whereas the other animals inoculated with CvHV2 assumingly were euthanized prior to sero-conversion. This is in line with previous inoculation experiments in reindeer with CvHV2 [36], in which a humoral immune response generally was not evident until days 8–10 p.i. Thus, the rapid onset of clinical signs after inoculation of CvHV2 in the conjunctiva indicates that a primary specific immune response will develop too late to be protective against disease in previously unexposed animals. This, and the observation that the seroprevalence among reindeer aged <1 year in Finnmark County, Norway, was only 8% as compared to 77% in adults (2004–2006) [26] suggests a rapid spread of the virus among young and immunologically naïve animals, sometimes resulting in outbreaks of IKC involving hundreds of animals [1, 18]. The hypothesis that stress causes reactivation of CvHV2 in latently infected animals [37] with subsequent viral shedding, exposing young and immunologically naïve animals to the virus, seems to be valid. During later stages of the disease, however, many different bacterial species, including *M. bovoculi*, have been isolated, but their role may be of a more opportunistic character, establishing infection and possibly being pathogenic once the mucosa is impacted by the CvHV2 infection. Based on the recent reports of the pathogenic properties of *M. bovoculi* in cattle, the pathogenic potential of the *M. bovoculi* isolates from reindeer should be further investigated.

No clinical signs were recorded in the three control animals, or in the left eye (control) of the inoculated reindeer (all groups). This lack of clinical signs in the left (control) eyes is in contrast to the detection of CvHV2-specific DNA from the left eyes of all CvHV2-inoculated animals from day 2 until the last sampling day, with the exceptions of animal R21 on day 6. At the same time, we were also unable to demonstrate viremia, since CvHV2-specific DNA was detected in a plasma sample from only one animal (R7) at one time point (day 2) during the experiment. Thus, these results indicate that the virus was able to spread from the inoculated conjunctiva to the conjunctival mucosa of the other eye within 2 days p.i. in all CvHV2- inoculated animals,

## Conclusions

Experimental inoculation of semi-domesticated reindeer revealed that CvHV2 is capable of inducing typical clinical signs of IKC within 2–7 days p.i., suggesting that the reindeer alphaherpesvirus, CvHV2, is the transmissible and causative agent of IKC in reindeer. The combined inoculation of CvHV2 and *M. bovoculi* did not change the onset, development, or the character of the clinical signs. Ocular inoculation of *M*. *bovoculi* alone did not produce clinical signs. *M. bovoculi* and other bacteria may, however, be important as opportunistic pathogens, especially during later stages of the disease. Based on the recent reports of the pathogenic properties of *M. bovoculi* in cattle [35, 38], the pathogenic potential of the *M. bovoculi* isolates from reindeer should be further investigated.
